# Health-Related Digital Engagement and Incident Stroke Among Older Adults: Prospective Cohort Study

**DOI:** 10.2196/93631

**Published:** 2026-07-06

**Authors:** Sufeng Zhou, Ruixuan Zhang, Haijun Qi, Hongbo Luo, Yuanlin Zou, Yuyang Zhou, Yi Zhou, Feiyan Yang

**Affiliations:** 1 Department of Cardiology, Central Hospital of Wuhan, Tongji Medical College, Huazhong University of Science and Technology, Shengli Street, Wuhan, Hubei, 430000, China, 86 02782211459; 2 College of Clinical Medicine, The First Bethune Hospital of Jilin University, Changchun, China

**Keywords:** stroke, older adults, health-related digital engagement, socioeconomic factors, longitudinal study, National Health and Aging Trends Study, NHATS

## Abstract

**Background:**

Most studies examining internet use and health outcomes in older adults rely on cross-sectional designs and binary exposure measures, which are insufficient to capture the multidimensional nature of health-related digital engagement over time. The high collinearity between digital engagement and socioeconomic factors makes it challenging to disentangle independent effects from marker effects. Longitudinal evidence linking health-related digital engagement to incident stroke remains limited.

**Objective:**

This study aimed to examine the longitudinal association between a composite Health-Related Digital Engagement Index (HDEI) and incident stroke among community-dwelling older adults, and to quantify the extent to which socioeconomic factors account for this association.

**Methods:**

This prospective cohort study used data from the National Health and Aging Trends Study, Waves 1‐10 (2011‐2020). The HDEI (range 0‐4) was constructed from 4 health-related internet behaviors assessed at baseline. The primary outcome was incident stroke ascertained by self- or proxy-reported physician diagnosis. Discrete-time hazard models with a complementary log-log link were fitted across 4 nested models, progressively adjusting for demographics, socioeconomic factors, chronic disease burden, functional disability, and social isolation.

**Results:**

Among 5384 participants followed for a median of 5 (IQR 2‐9) years, 472 incident stroke events were observed; 81.6% (4395/5384) had an HDEI score of 0, 10.5% (566/5384) had a score of 1, and 7.9% (423/5384) had a score of 2 or higher. In the unadjusted model, each 1-point increase in HDEI was associated with a lower risk of stroke (hazard ratio [HR] 0.76, 95% CI 0.66‐0.88; *P*<.001). After adjustment for age and sex, the association was attenuated but remained statistically significant (HR 0.84, 95% CI 0.72‐0.96; *P*=.01). After further adjustment for race or ethnicity, education, and household income, the association was no longer statistically significant (HR 0.92, 95% CI 0.79‐1.06; *P*=.23); fully adjusted analyses yielded similar results (HR 0.91, 95% CI 0.79‐1.05; *P*=.21). Subgroup patterns observed in demographically adjusted analyses were attenuated after socioeconomic adjustment, and no statistically significant interaction remained in the primary model 3 framework. Sensitivity analyses showed similar patterns of attenuation.

**Conclusions:**

Greater health-related digital engagement was associated with a lower risk of incident stroke in unadjusted and demographically adjusted models; however, this association was substantially attenuated and was no longer statistically significant after adjustment for socioeconomic factors. These findings are consistent with socioeconomic confounding and suggest that health-related digital engagement may, at least in part, reflect broader socioeconomic advantage among older adults. Future studies should further examine whether digital engagement has an independent role in stroke prevention beyond the socioeconomic and structural determinants of health.

## Introduction

Stroke is a leading cause of death and long-term disability worldwide. According to the World Stroke Organization 2025 Global Stroke Fact Sheet, stroke is the second leading cause of death and the third leading cause of disability globally, claiming approximately 7 million lives annually. It accounts for more than 160 million disability-adjusted life years and is estimated to impose annual economic losses of US $890 billion [1]. This burden falls disproportionately on older adults. Nearly 85% of strokes occur in individuals aged 65 years or older, and stroke incidence approximately doubles with each decade of age after 55 years [2,3]. In the United States, more than 3.69 million older adults died of stroke between 1999 and 2020; although the age-standardized mortality rate has declined over this period, substantial disparities persist across racial groups, sexes, and geographic regions [4]. A substantial body of evidence links socioeconomic status to both stroke incidence and poststroke outcomes; individuals with lower educational attainment, lower income, and limited economic resources face higher stroke risk and poorer functional recovery [5,6]. These findings have drawn increasing attention to the role of social determinants of health in stroke prevention and to persistent inequities in cerebrovascular outcomes [7].

The rapid advancement of digital health technologies is reshaping the delivery of health care services at both the system and individual levels. Digital health interventions include mobile health apps, telemedicine, access to online health information, and smart wearable devices, and are increasingly being integrated into chronic disease management and health promotion programs [8,9]. A systematic review and meta-analysis showed that these interventions improve self-management behaviors and physical activity levels in older adults with chronic diseases [10]. Evidence also suggests benefits for cognitive function and healthy aging [11]. Nevertheless, older adults have been slower than younger populations to adopt these technologies. Kebede et al [12] found that older adults experienced difficulties across multiple dimensions of digital technology engagement, including initial adoption and continuous daily use, and that these challenges were more pronounced among those from lower socioeconomic groups. Analysis of data from the US Health Information National Trends Survey further revealed that approximately one-quarter of older adults lacked digital access, and more than half did not use digital technology for health communication. This digital divide was significantly associated with poorer self-rated health [13].

Several longitudinal studies have investigated the association between internet use and health outcomes among older adults. Using data from the China Health and Retirement Longitudinal Study, Li et al [14] found that daily internet use was significantly associated with a lower risk of multiple chronic diseases, including stroke. Ren et al [15] pooled data from 4 cohorts in China, Mexico, the United States, and Europe and reported that internet users had significantly lower risks of diabetes, stroke, and all-cause mortality. Similarly, Nakagomi et al [16] found, in a cohort of older adults in Japan, that near-daily internet use was associated with improvements in multiple domains of social well-being and health behaviors. Studies using National Health and Aging Trends Study (NHATS) data have further shown that digital isolation, defined as nonuse of a cell phone, computer, email, or the internet, is significantly associated with increased risks of dementia, depression, and sleep disorders among older adults [17-19]. Despite this growing body of evidence, longitudinal data specifically linking digital engagement to cerebrovascular outcomes, including incident stroke, remain limited. Most existing studies rely on cross-sectional designs, which preclude the assessment of temporality, and use simple binary measures of internet use that do not adequately capture the multidimensional nature of health-related digital participation.

A more fundamental challenge is the strong collinearity between digital health engagement and socioeconomic status, which makes it difficult to determine whether digital engagement confers an independent protective effect or simply reflects socioeconomic advantage. The digital divide and health inequities are deeply intertwined through social determinants of health—individuals with lower income, lower educational attainment, and racial or ethnic minority backgrounds face both a higher risk of stroke and a greater likelihood of digital exclusion [6,7,13,20]. In the cardiovascular literature, adults aged 80 years and older have been reported to be 76% less likely to access telehealth services, and Black patients in the United States 36% less likely to use video-based medical visits [21]. Nevertheless, most studies examining the association between digital health engagement and chronic disease outcomes have not adequately addressed socioeconomic confounding, making it unclear whether the observed protective associations reflect a genuine benefit of digital engagement or the lower baseline risk of socioeconomically advantaged populations.

To address these knowledge gaps, the present study used baseline data from NHATS Wave 1 to construct a Health-Related Digital Engagement Index (HDEI), which comprehensively captures older adults’ use of the internet and digital technologies for health-related activities. Unlike previous studies that relied on a single binary indicator of internet use, the HDEI comprises 4 specific health-related online behaviors. NHATS is a nationally representative longitudinal study that has followed Medicare-enrolled adults aged 65 years and older annually since 2011 and has collected detailed data on functional status, cognition, technology use, and health outcomes [22,23].

This study had three objectives: (1) to examine the longitudinal association between HDEI and incident stroke in community-dwelling older adults, (2) to quantify the extent to which this association is explained by socioeconomic factors through sequential covariate adjustment, and (3) to assess potential effect modification across key demographic and clinical subgroups. The overarching aim was to determine whether health-related digital engagement represents an independent protective factor for stroke or primarily serves as a marker of socioeconomic advantage.

## Methods

### Study Design and Data Source

Data were obtained from the NHATS, a nationally representative longitudinal survey funded by the National Institute on Aging and conducted by the Johns Hopkins Bloomberg School of Public Health. NHATS uses a multistage sampling design to recruit adults aged 65 years and older, including both community-dwelling individuals and residents of residential care facilities, from the Medicare enrollment file [22]. Data are collected annually through computer-assisted personal interviews conducted by trained interviewers in participants’ homes, covering functional status, cognitive ability, technology use, health conditions, and socioeconomic characteristics. This study used Wave 1 (2011) as the baseline and Wave 10 (2020) as the end of follow-up, yielding a maximum follow-up period of 9 years.

### Study Population

**Figure 1.**
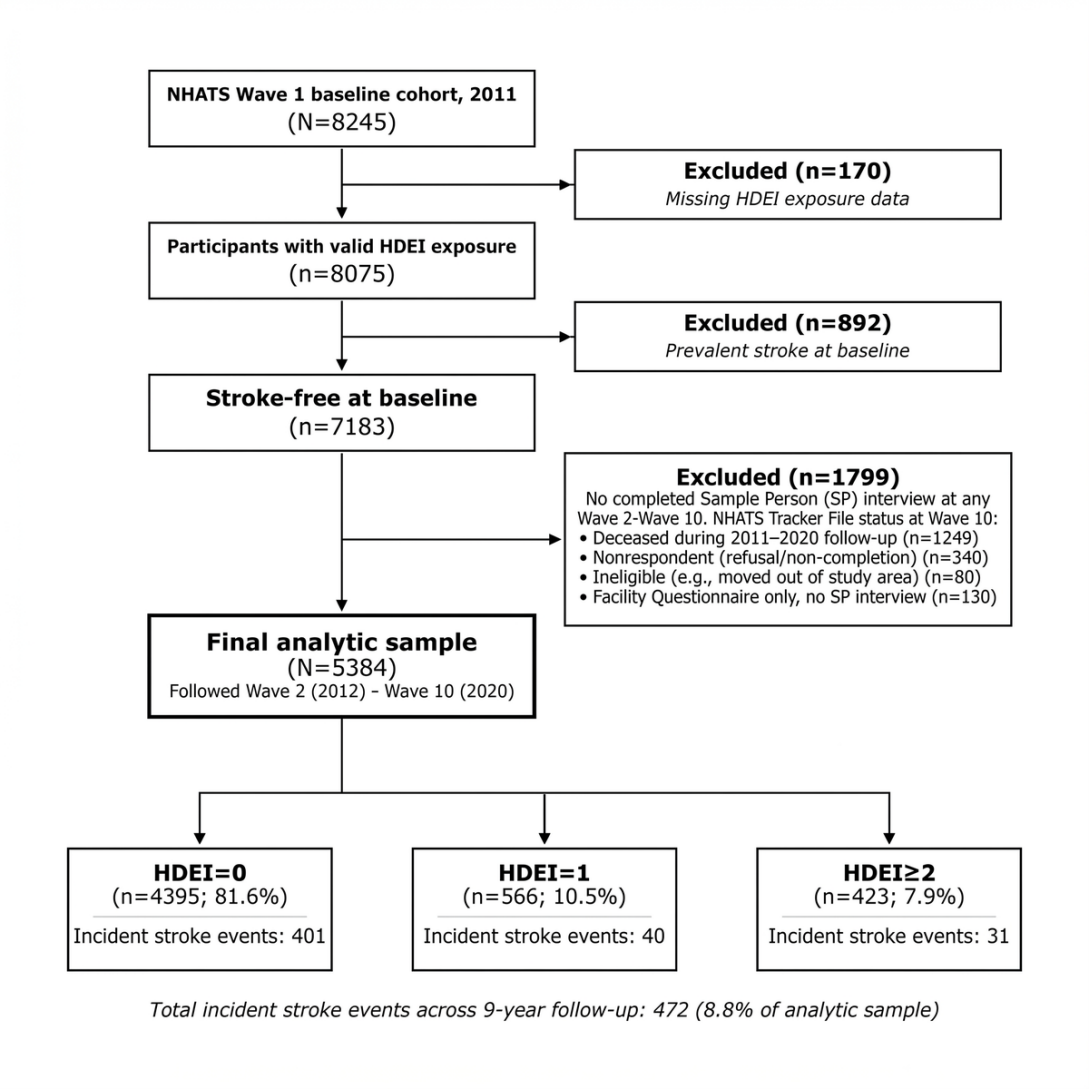
Flow diagram of participant selection from the National Health and Aging Trends Study, 2011‐2020. HDEI: Health-Related Digital Engagement Index; NHATS: National Health and Aging Trends Study.

The analytic cohort was derived from NHATS Wave 1 participants who were aged 65 years or older, had baseline information required to construct the HDEI, were free of self- or proxy-reported physician-diagnosed stroke at baseline, and had at least 1 completed Sample Person (SP) interview during follow-up from Wave 2 (2012) through Wave 10 (2020). We excluded participants with missing HDEI data, prevalent stroke at baseline, or no completed SP interview during follow-up. Participants without follow-up data were further classified using NHATS Tracker File status categories as deceased before follow-up, nonrespondents in all subsequent waves, ineligible in all subsequent waves, or having completed only Facility Questionnaire interviews. The unit of analysis was the individual NHATS SP at baseline; for discrete-time survival analyses, records were restructured into person-period observations across annual follow-up waves. The final analytic cohort included 5384 participants contributing 27,122 person-period records. The participant selection process is illustrated in Figure 1.

### Ethical Considerations

The original NHATS study protocol was approved by the Johns Hopkins Bloomberg School of Public Health Institutional Review Board (IRB 2083, first approved February 2011). The present secondary analysis was reviewed by the Ethics Committee of Wuhan Central Hospital and was granted an exemption from full ethical review on the grounds that the analysis involved only publicly accessible, deidentified data with no direct interaction with human participants; no case number was assigned for this exemption. All participants in the NHATS primary study provided written informed consent at the time of enrollment. The original IRB approval permits the use of deidentified data for secondary analyses; therefore, no additional informed consent was required for this study. All data were deidentified before public release by NHATS in accordance with established data governance procedures, ensuring that no individual participant can be identified from any data or results presented. No compensation was provided to research participants in connection with this secondary analysis. All figures, tables, and supplementary materials contain only aggregate or anonymized data; no individual participant can be identified from any image or data presented.

### Exposure Variable

The primary exposure was the HDEI, a composite measure derived from the NHATS Wave 1 questionnaire. The HDEI was designed to capture participants’ engagement in digital health care–related activities.

The index included 4 self-reported behaviors. Participants were asked whether they had used the internet in the past year to contact medical providers, manage Medicare or other health insurance matters, or search for health-related information. They were also asked whether they had ordered or refilled prescriptions online in the past month. Each item was coded as 1 for “yes” and 0 for “no.” The 4 items were summed to generate a total score ranging from 0 to 4, with higher scores indicating greater health-related digital engagement. A score of 0 indicated no engagement in any of the 4 behaviors, whereas a score of 4 indicated engagement in all 4 behaviors. The full wording of all items is provided in the NHATS codebook.

The HDEI was modeled in 2 complementary ways. First, it was analyzed as a continuous variable. In this model, the hazard ratio (HR) represented the association with each 1-point increase in the HDEI score. Second, participants were categorized into 3 groups according to their HDEI scores: 0, 1, and ≥2, with 0 used as the reference category. This categorical specification was used to examine potential nonlinear or threshold associations that might not be captured by the continuous specification.

The categorization into 0, 1, and ≥2 was prespecified based on the distribution of HDEI scores. Scores of 3 and 4 together accounted for only 3.5% (189/5384) of participants and were therefore combined with a score of 2 to ensure analytical stability. Although 3 items used a past-year recall window and one item—online prescription ordering or refilling—used a past-month recall window, all items were coded as binary indicators. Thus, the different recall periods affected the detection window for each item rather than its weight in the composite score. Any measurement error related to the different recall windows would be expected to be nondifferential with respect to future incident stroke and would therefore likely bias the estimates toward the null. Internal consistency was acceptable, with a Cronbach α of 0.68 and corrected item—total correlations ranging from 0.45 to 0.53. Equal weighting was applied, consistent with established composite indices of digital engagement.

### Outcome Variables

The primary outcome was incident stroke occurring during follow-up from Wave 2 through Wave 10, with Wave 1 serving as the baseline. Incident stroke was defined as the first wave at which a participant—or, where applicable, a proxy respondent—reported that a doctor had told them they had experienced a stroke since the previous interview. A first-event design was applied; participants were classified as having experienced the outcome at the wave of first report and were not followed further, while those who never reported stroke were censored at their last available follow-up interview.

### Covariates

Guided by previous literature and a directed acyclic graph (DAG)–based causal framework, covariates were grouped into 4 conceptual blocks according to their presumed roles in the causal pathway between HDEI and incident stroke. Age and sex were considered demographic confounders because they may influence both digital health engagement and baseline stroke risk. Age was categorized into 5-year intervals—65‐69, 70‐74, 75‐79, 80‐84, 85‐89, and ≥90 years—and entered as a categorical variable to allow for a nonlinear association with stroke risk. Sex was included as a binary variable [24].

Race or ethnicity, educational attainment, and household income were considered socioeconomic confounders because they may shape access to digital health services and influence cerebrovascular risk through health care access, health literacy, and material resources. Race or ethnicity was categorized as non-Hispanic White, non-Hispanic Black, non-Hispanic Other, and Hispanic. Educational attainment was categorized as high school or less, some college, and bachelor’s degree or higher. Annual household income was categorized as <US $25,000, US $25,000-US $49,999, US $50,000-US $74,999, and ≥US $75,000 [6,25]. Participants with missing, “don’t know,” or refused income responses were retained as a separate category to preserve sample size.

Baseline health and social-context factors were considered additional confounders and included in an extended model to assess the robustness of the primary estimate adjusted for socioeconomic factors. These factors included chronic disease burden, defined as the number of 9 self-reported physician-diagnosed conditions at baseline—heart attack, heart disease, high blood pressure, arthritis, osteoporosis, diabetes, lung disease, dementia or Alzheimer disease, and cancer—and modeled as a continuous variable ranging from 0 to 9; and an activities of daily living (ADL) disability index, modeled as a continuous variable [24,26]. A social isolation index, modeled as a continuous variable ranging from 0 to 6, was constructed from NHATS Wave 1 items on living alone, having a limited social network, and not participating in 4 in-person social activities during the past month—visiting friends or family, attending religious services, participating in club or group meetings, and volunteering. This index was included to control for conventional offline social isolation and to distinguish it from the digital divide captured by the HDEI [27,28].

Potential effect modification by age, sex, race or ethnicity, and education was assessed separately using prespecified subgroup and interaction analyses. No postbaseline variables, descendants of the exposure, or variables considered colliders were intentionally adjusted for in the primary model. The DAG used to guide covariate selection is presented in Multimedia Appendix 1.

### Statistical Analysis

Baseline characteristics were described by HDEI category (0, 1, and ≥2) and reported as means (SD) or frequencies (%). Between-group differences were assessed using standardized mean differences (SMDs), with SMD>0.10 indicating a meaningful imbalance. To evaluate potential selection bias from loss to follow-up, an attrition analysis compared retained and excluded participants on all baseline characteristics using SMD, independent-samples *t* tests, and chi-squared tests.

Incident stroke risk was estimated using discrete-time hazard models with a complementary log-log link. Data were restructured into person-period format, with follow-up wave as a time-varying intercept. Wave 1 analytic weights were applied throughout to yield nationally representative estimates, and robust variance was used to account for within-person correlation. In addition, the primary sampling unit and stratum identifiers provided in the NHATS public-use files were incorporated in all models to account for the multistage cluster sampling design and obtain correct SEs. Moreover, 4 nested models were fitted: model 1 (unadjusted), model 2 (age and sex), model 3 (further adjusted for race or ethnicity, education, and household income), and model 4 (further adjusted for chronic disease burden, ADL disability index, and social isolation index). Missing or refused income responses were retained as a separate indicator category. Participants with missing race or ethnicity or educational attainment were excluded from models that included these covariates. Thus, models 3 and 4 excluded 59 participants (including overlap) with missing race or ethnicity or educational attainment, while missing household income was retained as a separate category. The HDEI was entered as both a continuous variable and an ordinal categorical variable, with a *P* for trend obtained from the ordinal specification.

To assess within-model multicollinearity, variance inflation factors (VIFs) were calculated for all predictors in models 2, 3, and 4 using the categorical dummy-variable specification described above; the condition number of the fully adjusted design matrix was computed as a supplementary diagnostic. The proportional hazards assumption was tested by including a multiplicative HDEI×follow-up–wave interaction term in each primary model; a nonsignificant interaction indicates that the HDEI effect was stable across the observation period.

Prespecified subgroup analyses were performed within both the model 2 and model 3 frameworks, with model 3 (socioeconomic-adjusted) serving as the primary inferential framework, stratified by sex, age (<80 vs ≥80 y), education, and race or ethnicity; interaction was assessed via multiplicative product terms. Furthermore, two sensitivity analyses were conducted: (1) exclusion of Wave 2 events to minimize reverse-causation bias, and (2) substitution of cell phone use as an alternative technology-exposure variable. In addition, three extra sensitivity analyses addressed potential threats to inference: (1) a discrete-time competing-risks analysis using multinomial logistic regression on the same person-period dataset, with a 3-level outcome at each person-wave (no event, incident stroke, or death, with death ascertained from the NHATS Tracker File), to verify robustness to the cause-specific censoring assumption; (2) a disaggregated chronic-disease specification entering each of the 9 baseline conditions as separate binary covariates (model 4b), to verify robustness to the composite chronic-disease index; and (3) refitting models 1‐3 after excluding the 215 participants (4%) with self-reported physician-diagnosed dementia at baseline, to address the possibility that severe baseline cognitive impairment confounds the HDEI-stroke association.

Unadjusted cumulative incidence curves by HDEI category were estimated using the Kaplan-Meier method, and between-group differences were evaluated with the log-rank test for descriptive purposes only. To assess robustness to the missing-income indicator approach, we performed multiple imputation by chained equations (MICE; m=20) for missing baseline covariates. Each completed dataset was transformed into the same person-period structure used in the primary analysis, and models 3 and 4 were re-estimated using survey-weighted discrete-time hazard models with a complementary log-log link. Estimates were pooled on the log-HR scale using Rubin’s rules and reported as pooled HRs with 95% CIs. To address covariate imbalance across HDEI groups, we conducted an inverse probability of treatment weighting (IPTW) sensitivity analysis using multinomial propensity scores. Stabilized IPTW weights were trimmed at the 99th percentile, combined with NHATS sampling weights, and applied in survey-weighted marginal structural discrete-time hazard models; covariate balance was assessed using the maximum absolute SMD across HDEI group contrasts.

All tests were 2-sided; *P*<.05 was considered statistically significant. Analyses were performed in Python (version 3.12; Python Software Foundation) and R (version 4.4.1; R Core Team).

## Results

### Study Population and Baseline Characteristics

As illustrated in Figure 1, the Wave 1 baseline cohort comprised 8245 participants; after excluding those with missing HDEI data (n=170), prevalent stroke (n=892), and no follow-up data (n=1799), the analytic cohort included 5384 participants, contributing 27,122 person-period records across Waves 2‐10, with 472 incident stroke events over a median follow-up of 5 (IQR 2‐9) years. Compared with retained participants, those excluded due to missing follow-up were older, less educated, had lower household income, and had substantially lower baseline HDEI scores (mean 0.14, SD 0.52 vs mean 0.31, SD 0.76; SMD 0.264; Multimedia Appendix 2).

Baseline characteristics by HDEI category are presented in Table 1. Participants with higher HDEI scores were younger (65‐69 y: 168/423, 39.7% in HDEI≥2 vs 723/4395, 16.5% in HDEI=0; SMD 0.536), more likely to be non-Hispanic White (364/423, 86.1% vs 2891/4395, 65.8%; SMD 0.488), more educated (bachelor’s degree or above: 258/423, 61% vs 940/4395, 21.4%; SMD 1.028), and had higher household income (≥$75,000: 122/423, 28.8% vs 270/4395, 6.1%; SMD 0.625). Higher HDEI was also associated with lower ADL disability (mean 0.50, SD 1.09 vs mean 0.91, SD 1.55; SMD 0.321), lower social isolation (mean 2.25, SD 1.24 vs mean 2.73, SD 1.21; SMD 0.385), and greater cell phone use (400/423, 94.6% vs 3031/4395, 69%; SMD 0.702). Differences in chronic disease burden were modest (SMD 0.082). Collectively, these patterns indicate a pronounced digital divide in which higher health-related digital engagement was strongly codistributed with socioeconomic advantage.

**Table 1.** Baseline characteristics of participants by Health-Related Digital Engagement Index. Data are presented as n (%) for categorical variables and mean (SD) for continuous variables unless otherwise indicated. SMD^a^ was computed as the maximum pairwise standardized mean difference across the three HDEI groups.

Characteristic	HDEI^b^=0 (n=4395)	HDEI=1 (n=566)	HDEI≥2 (n=423)	Overall (n=5384)	SMD
Age group (y), n (%)					
65‐69	723 (16.5)	180 (31.8)	168 (39.7)	1071 (19.9)	0.536
70‐74	892 (20.3)	156 (27.6)	107 (25.3)	1155 (21.5)	0.170
75‐79	921 (21)	110 (19.4)	66 (15.6)	1097 (20.4)	0.139
80‐84	898 (20.4)	73 (12.9)	57 (13.5)	1028 (19.1)	0.203
85‐89	565 (12.8)	37 (6.5)	22 (5.2)	624 (11.6)	0.269
≥90	396 (9)	10 (1.8)	3 (0.7)	409 (7.6)	0.393
Female, n (%)	2650 (60.3)	304 (53.7)	192 (45.4)	3146 (58.4)	0.302
Race or ethnicity, n (%)					
White, non-Hispanic	2891 (65.8)	483 (85.3)	364 (86.1)	3738 (69.4)	0.488
Black, non-Hispanic	1050 (23.9)	53 (9.4)	41 (9.7)	1144 (21.2)	0.398
Other, non-Hispanic	125 (2.8)	11 (1.9)	7 (1.7)	143 (2.7)	0.080
Hispanic	288 (6.6)	15 (2.7)	9 (2.1)	312 (5.8)	0.218
Missing	41 (1)	4 (0.7)	2 (0.5)	47 (0.9)	—^c^
Education^d^, n (%)					
≤High school	2582 (58.7)	135 (23.9)	62 (14.7)	2779 (51.6)	1.028
Some college	829 (18.9)	129 (22.8)	101 (23.9)	1059 (19.7)	0.123
Bachelor’s or higher	940 (21.4)	300 (53)	258 (61)	1498 (27.8)	0.879
Missing	44 (1)	2 (0.4)	2 (0.5)	48 (0.9%)	—
Household income, n (%)					
Missing, do not, know, or refused	1926 (43.8)	217 (38.3)	127 (30)	2270 (42.2)	—
<US $25,000	1261 (28.7)	74 (13.1)	39 (9.2)	1374 (25.5)	0.513
US $25,000-US $49,999	650 (14.8)	95 (16.8)	63 (14.9)	808 (15)	0.055
US $50,000-US $74,999	288 (6.6)	61 (10.8)	72 (17)	421 (7.8)	0.329
≥US $75,000	270 (6.1)	119 (21)	122 (28.8)	511 (9.5)	0.625
Chronic disease burden^e^, mean (SD)					
Index score	2.39 (1.47)	2.33 (1.43)	2.27 (1.42)	2.37 (1.46)	0.082
Heart attack	585 (13.3)	61 (10.8)	51 (12.1)	697 (12.9)	0.078
Heart disease	737 (16.8)	91 (16.1)	83 (19.6)	911 (16.9)	0.093
High blood pressure	2907 (66.1)	350 (61.8)	273 (64.5)	3530 (65.6)	0.089
Arthritis	2416 (55.0)	313 (55.3)	207 (48.9)	2936 (54.5)	0.128
Osteoporosis	885 (20.1)	115 (20.3)	66 (15.6)	1066 (19.8)	0.123
Diabetes	1090 (24.8)	111 (19.6)	85 (20.1)	1286 (23.9)	0.125
Lung disease	598 (13.6)	90 (15.9)	68 (16.1)	756 (14.0)	0.070
Dementia or Alzheimer disease	210 (4.8)	4 (0.7)	1 (0.2)	215 (4.0)	0.294
Cancer	1058 (24.1)	182 (32.2)	125 (29.6)	1365 (25.3)	0.181
ADL^f^ disability^g^, mean (SD)					
Index score	0.91 (1.55)	0.48 (1.04)	0.50 (1.09)	0.83 (1.48)	0.321
Bathing	739 (16.8)	40 (7.1)	32 (7.6)	811 (15.1)	0.304
Dressing	796 (18.1)	58 (10.2)	50 (11.8)	904 (16.8)	0.227
Eating	313 (7.1)	14 (2.5)	9 (2.1)	336 (6.2)	0.239
Getting around inside	868 (19.7)	62 (11.0)	45 (10.6)	975 (18.1)	0.256
Toileting	403 (9.2)	25 (4.4)	18 (4.3)	446 (8.3)	0.197
Transferring	871 (19.8)	75 (13.3)	56 (13.2)	1002 (18.6)	0.178
Social isolation^h^, mean (SD)					
Index score	2.73 (1.21)	2.36 (1.13)	2.25 (1.24)	2.65 (1.21)	0.385
Living alone	2989 (68.0)	449 (79.3)	340 (80.4)	3778 (70.2)	0.285
Limited social network	311 (7.1)	22 (3.9)	15 (3.5)	348 (6.5)	0.158
Nonparticipation, mean (SD)	1.98 (1.10)	1.52 (1.07)	1.41 (1.14)	1.89 (1.12)	0.502
Cell phone use, n (%)	3031 (69)	528 (93.3)	400 (94.6)	3959 (73.5)	0.702
Incident stroke, n (%)	401 (9.1)	40 (7.1)	31 (7.3)	472 (8.8)	—
Follow-up waves, median (IQR)	5 (2–9)	8 (2–9)	9 (3–9)	5 (2–9)	—

a SMD: standardized mean difference.

b HDEI: Health-Related Digital Engagement Index.

c Not available.

d Education data were missing for 48 participants (0.9% of the analytic sample), and race or ethnicity data were missing for 47 participants (0.9%). Participants with missing race or ethnicity or educational attainment were excluded from regression models that included these covariates; missing, “don’t know,” or refused household income responses were retained as a separate category.

e The chronic disease burden index was calculated as the sum of 9 self-reported physician-diagnosed conditions (heart attack, heart disease, high blood pressure, arthritis, osteoporosis, diabetes, lung disease, dementia or Alzheimer disease, and cancer); range, 0 to 9.

f ADL: activities of daily living.

g The ADL disability index was calculated as the sum of 6 self-reported limitations in activities of daily living (bathing, dressing, eating, getting around inside, toileting, and transferring); range, 0 to 6.

h The social isolation index was calculated as the sum of living alone (0 or 1), having a limited social network (0 or 1), and number of social activities not participated in (0 to 4); range, 0 to 6.

### Association Between HDEI and Incident Stroke

Results from the 4 nested models are presented in Table 2. In the unadjusted model (model 1), each 1-point increase in HDEI was associated with a lower risk of stroke (HR 0.76, 95% CI 0.66‐0.88; *P*<.001). In the categorical analysis, both the HDEI=1 group (HR 0.64, 95% CI 0.46‐0.88) and the HDEI≥2 group (HR 0.63, 95% CI 0.44‐0.92) had significantly lower risks of stroke than the reference group (*P* for trend=.001). After adjustment for age and sex (model 2), the HR for the continuous HDEI attenuated to 0.84 (95% CI 0.72‐0.96; *P*=.01). The association for the HDEI=1 group remained statistically significant (HR 0.71, 95% CI 0.51‐0.98; *P*=.04), whereas that for the HDEI≥2 group did not (HR 0.75, 95% CI 0.52‐1.09; *P*=.13).

**Table 2.** Association between Health-Related Digital Engagement Index and incident stroke: discrete-time hazard models with sequential covariate adjustment.

Model and exposure	n^a^	Events^a^	HR^b^ (95% CI)	*P* value	*P* for trend
Model 1: unadjusted (+wave)^c^	5384	472			.001
Per 1-point increase			0.76 (0.66‐0.88)	<.001	
HDEI^d^=0 (reference)			1.00^e^	—^f^	
HDEI=1			0.64 (0.46‐0.88)	.006	
HDEI≥2			0.63 (0.44‐0.92)	.02	
Model 2: age and sex^g^	5384	472			.03
Per 1-point increase			0.84 (0.72‐0.96)	.01	
HDEI=0 (reference)			1.00^e^	—	
HDEI=1			0.71 (0.51‐0.98)	.04	
HDEI≥2			0.75 (0.52‐1.09)	.13	
Model 3: race or ethnicity, education, and income^h^	5325	465			.45
Per 1-point increase			0.92 (0.79‐1.06)	.23	
HDEI=0 (reference)			1.00^e^	—	
HDEI=1			0.82 (0.58‐1.15)	.24	
HDEI≥2			0.93 (0.63‐1.37)	.72	
Model 4: chronic disease, ADL^i^, and social isolation^j^	5325	465			.45
Per 1-point increase			0.91 (0.79‐1.05)	.21	
HDEI=0 (reference)			1.00^e^	—	
HDEI=1			0.83 (0.59‐1.16)	.28	
HDEI≥2			0.93 (0.63‐1.36)	.70	

a n and events refer to model-specific sample sizes and event counts.

b HR: hazard ratio.

c Model 1: unadjusted (wave indicators only).

d HDEI: Health-Related Digital Engagement Index.

e 95% CI not applicable.

f Not applicable.

g Model 2: adjusted for age (6 categories) and sex.

h Model 3: further adjusted for race or ethnicity, education, and household income (missing income retained as a separate category).

i ADL: activities of daily living.

j Model 4: further adjusted for chronic disease burden, activities of daily living disability index, and social isolation index.

**Figure 2.**
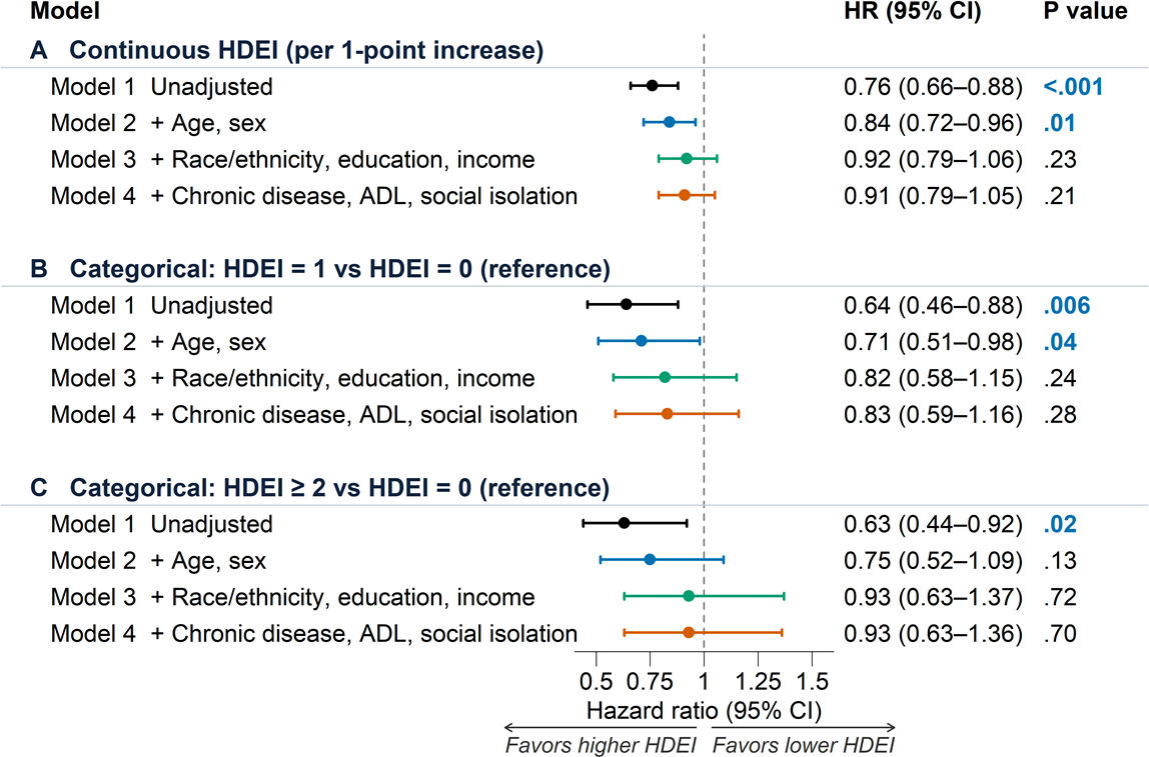
Association between Health-Related Digital Engagement Index (HDEI) and risk of incident stroke. (A) The upper panel shows continuous HDEI (per 1-unit increase); (B) the middle and (C) lower panels show categorical HDEI (HDEI=1 and HDEI≥2, respectively, vs HDEI=0 as the reference). Model 1 was unadjusted. Model 2 adjusted for age and sex. Model 3 additionally adjusted for race or ethnicity, education, and household income. Model 4 further adjusted for chronic disease burden, activities of daily living disability, and social isolation. Bold blue *P* values indicate statistical significance at the .05 level. Error bars represent 95% CIs. HDEI: Health-Related Digital Engagement Index; HR: hazard ratio; ADL: activities of daily living.

Further adjustment for race or ethnicity, education, and household income (model 3) markedly attenuated the association to nonsignificance (continuous HDEI: HR 0.92, 95% CI 0.79‐1.06; *P*=.23; HDEI=1: HR 0.82 CI 0.58-1.15; *P*=.25; HDEI≥2: HR 0.93, 95% CI 0.63-1.37; *P*=.72; *P* for trend=.45). The fully adjusted model (model 4) yielded nearly identical results (continuous HDEI: HR 0.91, 95% CI 0.79‐1.05; *P*=.21), indicating that additional adjustment for chronic disease burden, ADL disability, and social isolation produced no further attenuation. The attenuation pattern across all 4 models and 3 exposure parameterizations is illustrated in Figure 2.

Model diagnostics confirmed the stability of all estimates. VIFs were below 1.70 for every predictor across models 2, 3, and 4 (maximum VIF=1.68, age group 75‐79 in model 4; VIF for HDEI=1.20 in model 4), and the condition number of the fully adjusted design matrix was 29.3, both well within acceptable limits (refer to Multimedia Appendix 3 for full VIF table). The proportional hazards assumption was not violated: the HDEI×follow-up-wave interaction term was nonsignificant across all primary models (model 4: coefficient=−0.007; *P*=.80), indicating that the effect of HDEI on stroke hazard remained stable throughout the 9-year follow-up period.

In Figure 2 and Table 2, HRs were estimated using discrete-time hazard models with a complementary log-log link. The HDEI was modeled both as a continuous variable (per 1-point increase) and as a categorical variable (0 [reference], 1, and ≥2). *P* for trend was obtained by entering HDEI categories as a single ordinal term. Models 3 and 4 excluded 59 participants with missing race or ethnicity or educational attainment, and missing household income responses were retained as a separate category.

### Unadjusted Cumulative Stroke Incidence

**Figure 3.**
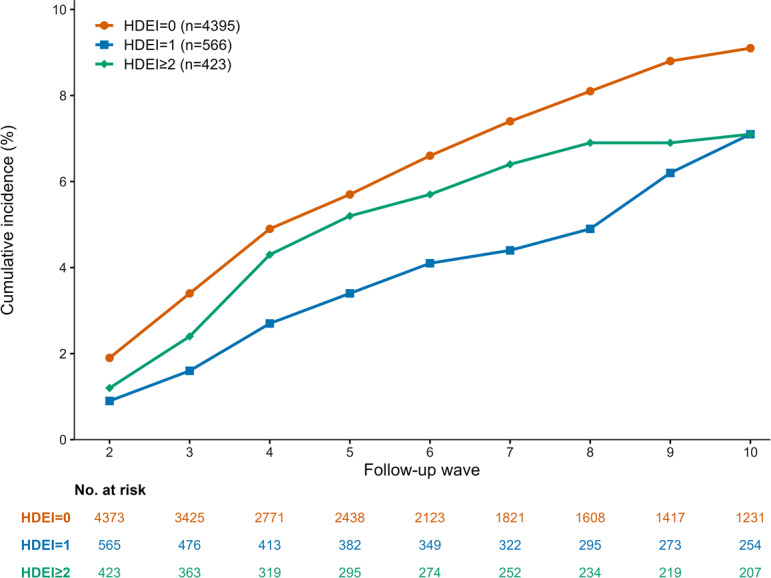
Unadjusted Kaplan-Meier cumulative incidence curves for incident stroke by Health-Related Digital Engagement Index (HDEI) category. Unadjusted Kaplan-Meier cumulative incidence curves are shown by HDEI category: HDEI=0, HDEI=1, and HDEI ≥2. Between-group differences were evaluated using the log-rank test for descriptive purposes only. Numbers at risk at each wave are shown below the figure. Participants were censored at the wave of first stroke or at their last observed wave. Because the Kaplan-Meier method treats deaths before stroke as non-informative censoring, these unadjusted estimates may overestimate the true cumulative incidence of stroke in the presence of the competing risk of death. HDEI: Health-Related Digital Engagement Index.

Unadjusted cumulative incidence curves showed that differences in stroke incidence across HDEI groups emerged early in follow-up and persisted throughout the observation period. Cumulative incidence was highest in the HDEI=0 group, reaching approximately 9% by Wave 10, compared with approximately 7% in both the HDEI=1 and HDEI≥2 groups (Figure 3). These curves represent unadjusted descriptive estimates and should not be interpreted as evidence of a causal association.

### Subgroup Analyses

Prespecified subgroup analyses are presented in Table 3, with the model 3 (socioeconomic-adjusted) framework serving as the primary inferential framework and model 2 estimates retained for transparency. Under model 3, no subgroup HR reached statistical significance at the 0.05 threshold, and no interaction term provided evidence of effect modification. By sex, the HDEI-stroke association was numerically more protective among male (HR 0.83, 95% CI 0.66‐1.05; *P*=.12) than among female (HR 1.02, 95% CI 0.84‐1.24; *P*=.85); the formal interaction test was not significant (*P* for interaction=.16). By age, the association was directionally consistent across both strata but reached significance in neither under model 3 (HR 0.92, 95% CI 0.78‐1.09; *P*=.33) among participants younger than 80 years, and HR 0.78 (95% CI 0.55‐1.09; *P*=.14) among those aged ≥80 years (*P* for interaction=.22). A similar pattern was observed for education; the lower-education group (≤high school) showed a nonsignificant protective trend (HR 0.74, 95% CI 0.48‐1.12; *P*=.16), while the higher-education group did not (HR 0.95, 95% CI 0.81‐1.11; *P*=.52; *P* for interaction=.22). When stratified by race or ethnicity, the association did not reach significance in either stratum under model 3 (non-Hispanic White: HR 0.91, 95% CI 0.78‐1.07; *P*=.27; non-White: HR 0.87, 95% CI 0.58‐1.30, *P*=.50; *P* for interaction=.52). The directional patterns observed in the unadjusted model 2 estimates—including the protective association among men and the numerically protective associations in the ≥80 age stratum—were attenuated to nonsignificance after adjustment for socioeconomic factors (Table 3). We therefore interpret all subgroup findings as exploratory and hypothesis-generating, consistent with the primary analysis showing that socioeconomic factors largely account for the apparent HDEI-stroke association.

**Table 3.** Subgroup analyses of the association between Health-Related Digital Engagement Index and incident stroke.

Subgroup	n^a^	Events^a^	Model 2^b^		Model 3^e^		
			HR^c^^d^ (95% CI)	*P* value	HR (95% CI)	*P* value	*P* for interaction^f^
Overall	5384	472	0.84 (0.72‐0.96)	.01	0.92 (0.79‐1.06)	.23	—^g^
Sex							.16
Male	2238	189	0.74 (0.59‐0.91)	.006	0.83 (0.66‐1.05)	.12	
Female	3146	283	0.95 (0.79‐1.15)	.62	1.02 (0.84‐1.24)	.85	
Age (y)							.22
<80	3323	274	0.84 (0.72‐0.98)	.03	0.92 (0.78‐1.09)	.33	
≥80	2061	198	0.70 (0.50‐0.97)	.03	0.78 (0.55‐1.09)	.14	
Education							.22
≤High school	2779	260	0.68 (0.45‐1.04)	.08	0.74 (0.48‐1.12)	.16	
>High school	2557	206	0.91 (0.78‐1.07)	.27	0.95 (0.81‐1.11)	.52	
Race or ethnicity							.52
Non-Hispanic White	3738	305	0.88 (0.75‐1.03)	.11	0.91 (0.78‐1.07)	.27	
Non-White	1599	162	0.75 (0.51‐1.10)	.14	0.87 (0.58‐1.30)	.50	

a n and Events refer to subgroup counts in the full analytic cohort before model-specific exclusion of participants with missing race or ethnicity or educational attainment.

b Model 2: adjusted for age (6 categories) and sex.

c HR: hazard ratio.

d HRs are per 1-point increase in Health-Related Digital Engagement Index (continuous, range 0‐4).

e Model 3 (primary inferential framework): further adjusted for race or ethnicity, educational attainment, and household income (missing income retained as a separate category). Under Model 3, no subgroup hazard ratio or interaction term reached statistical significance at the 0.05 threshold. Model 3 estimates excluded some participants as described in the Statistical Analysis section.

f *P* values for interaction were obtained from Wald tests of the Health-Related Digital Engagement Index×subgroup interaction term within the Model 3 framework.

g Not applicable.

### Sensitivity Analyses

Sensitivity analyses generally supported the attenuation pattern observed in the primary analysis, although the IPTW findings were interpreted cautiously because covariate balance was not fully achieved after weighting. Excluding events reported at Wave 2 to reduce potential reverse-causation bias did not materially alter the results. The association remained significant after demographic adjustment (model 2: HR 0.82, 95% CI 0.70‐0.95; *P*=.01) but was attenuated to nonsignificance after full adjustment (model 4: HR 0.91, 95% CI 0.78‐1.06; *P*=.24). This finding suggests that the attenuation pattern was not primarily driven by events occurring shortly after baseline.

Substituting cell phone use as an alternative technology exposure yielded a similar attenuation pattern. The association was significant after demographic adjustment (model 2: HR 0.77, 95% CI 0.62‐0.95; *P*=.01) but was attenuated after full adjustment (model 4: HR 0.90, 95% CI 0.73‐1.12; *P*=.36). This result suggests that the observed pattern was not specific to health-related digital engagement but reflected broader socioeconomic patterning in technology use. Full results of the reverse-causation exclusion and alternative exposure analyses are presented in Multimedia Appendix 4.

The MICE sensitivity analysis, re-estimated using the same survey-weighted discrete-time hazard framework as the primary analysis, produced results consistent with those of the primary missing-indicator analysis. In model 3, the pooled HR for a 1-point increase in HDEI was 0.93 (95% CI 0.80‐1.07; *P*=.30), and the corresponding pooled HR in model 4 was 0.92 (95% CI 0.80‐1.06; *P*=.27; Multimedia Appendix 5). The IPTW sensitivity analysis, re-estimated as a survey-weighted marginal structural discrete-time hazard model, yielded a nominally inverse association for a 1-point increase in HDEI (HR 0.84, 95% CI 0.71‐1.00; *P*=.048). However, covariate balance was not fully achieved after weighting for several socioeconomic and functional variables. In a doubly robust specification that additionally adjusted for the same covariates, the association was attenuated to nonsignificance (HR 0.86, 95% CI 0.73‐1.03; *P*=.10; Multimedia Appendix 6). Therefore, the IPTW results were interpreted as supportive but not definitive evidence under an alternative weighting approach.

Three additional prespecified sensitivity analyses further supported the stability of the model 3 estimate. A discrete-time competing-risks analysis was conducted using multinomial logistic regression on the same person-period dataset, with death treated as a competing event and nonstroke loss to follow-up treated as noninformative censoring. This analysis yielded a model 3 relative risk ratio for stroke of 0.92 (95% CI 0.79‐1.07; *P*=.27), closely matching the primary discrete-time model 3 estimate (Multimedia Appendix 7). Replacing the composite chronic disease index with 9 separate binary indicators for individual baseline conditions yielded an HR for HDEI of 0.91 (95% CI 0.79‐1.05; *P*=.18; Multimedia Appendix 8). After excluding 215 participants (4%) with self-reported physician-diagnosed dementia at baseline, the model 3 HR was 0.91 (95% CI 0.78‐1.06; *P*=.20). This result suggests that the primary findings were not driven by reverse causation due to baseline cognitive impairment (Multimedia Appendix 9). Across these additional sensitivity analyses, the HDEI estimates ranged from 0.91 to 0.92 and remained nonsignificant.

## Discussion

### Principal Findings

In this prospective cohort of community-dwelling older adults followed for up to 9 years, higher health-related digital engagement was associated with lower stroke incidence before socioeconomic adjustment but not after. The stepwise attenuation of the HR across nested models—from HR 0.76 in the unadjusted model to HR 0.84 after demographic adjustment and HR 0.92 after socioeconomic adjustment—is consistent with substantial socioeconomic confounding and suggests that the crude inverse association may largely reflect the broader socioeconomic profile of digitally engaged older adults. The stability of the estimate between models 3 and 4 provides limited evidence that the measured baseline health-status variables explain the association beyond socioeconomic adjustment; however, formal mediation cannot be inferred from these models. Consistent with the DAG (Multimedia Appendix 1), chronic disease burden, ADL disability, and social isolation were conceptualized as baseline confounders rather than intermediaries, given that all 3 were measured contemporaneously with the exposure and plausibly share common causes with both HDEI and stroke risk.

These findings are consistent with the broader evidence base positioning digital exclusion as a manifestation of socioeconomic disadvantage rather than an independent health determinant. Exclusion from digital technology has been associated with accelerated functional decline and greater psychological distress, including lower self-rated health, contributing to reduced life expectancy among those with limited access to information technology [29]. In the United States, the digital divide among older adults has narrowed gradually over time but remains closely linked to socioeconomic disparities in health [13]. This study extends this evidence to incident stroke, suggesting that the digital divide and cerebrovascular risk share common socioeconomic determinants.

### Comparison With Previous Work

The exposure in this study—the HDEI—captures health-specific digital behaviors, encompassing online interaction with medical providers, insurance management, health information seeking, and prescription management, rather than general internet access or passive web browsing. This distinction is important; previous research has shown that, under equivalent conditions of access, digital skills, health literacy, and perceived usability of digital health tools are key determinants of whether technology translates into measurable health benefits [30]. Although the independent association between HDEI and stroke did not survive socioeconomic adjustment, this does not preclude health-related digital engagement from contributing to stroke prevention through indirect pathways. Improved medication adherence, more consistent engagement with chronic disease management programs, and enhanced health information access may all represent plausible mechanisms; however, these pathways are deeply entangled with education, income, and health literacy, making their isolation from socioeconomic confounding difficult in observational data.

Socioeconomic advantage may reduce stroke risk through several interrelated mechanisms in which digital engagement serves as one expression of a broader structural advantage rather than an independent causal pathway. First, individuals with greater economic and social resources and higher digital capability may achieve earlier identification and better management of modifiable stroke risk factors, including hypertension, diabetes, dyslipidemia, and atrial fibrillation—risk factor control that is broadly accepted to reduce the population burden of stroke [8]. Second, digital engagement may facilitate access to health care, support treatment continuity, and enable medication adherence for patients requiring ongoing chronic disease management. The rapid expansion of telemedicine following the COVID-19 pandemic has amplified the consequences of differential digital capacity, with lower-HDEI individuals facing greater barriers to care and prolonged exposure to uncontrolled risk factors [31]. Third, digital tools may operate through psychosocial pathways by reducing social isolation and strengthening supportive networks, given established associations between loneliness and adverse cardiovascular and cerebrovascular outcomes [28,32,33]. However, the absence of further attenuation when adjusting for social isolation in model 4 relative to model 3 suggests that psychosocial pathways are unlikely to be the primary mechanism in this cohort.

### Sensitivity Analyses and Methodological Considerations

Sensitivity analyses generally supported the attenuation pattern observed in the main analysis. Excluding Wave 2 events produced similar findings, suggesting that early postbaseline events were unlikely to fully explain the observed association. Substituting cell phone use as an alternative technology exposure also yielded a comparable attenuation pattern, indicating that the crude association may reflect broader socioeconomic patterning in technology use rather than a specific effect of health-related digital engagement. The MICE analysis, re-estimated using the same discrete-time survival framework as the primary analysis, produced similar estimates, suggesting that the findings were not driven by the missing-income indicator approach. The IPTW analysis yielded directionally supportive results. However, because covariate balance was not fully achieved after weighting and the association was attenuated to nonsignificance in the doubly robust specification, these results should be interpreted cautiously rather than as definitive causal evidence. Methodologically, survey-weighted discrete-time hazard models with a complementary log-log link and wave-specific intercepts were appropriate for the annually assessed, interval-censored outcome structure of NHATS, whose instruments have established reliability and validity for long-term follow-up [22,34,35]. Although the HDEI-stroke association appeared numerically stronger among males than among females, the sex interaction was not statistically significant (*P*=.16). This subgroup pattern should therefore be considered hypothesis-generating.

### Implications for Practice and Policy

These findings suggest that the digital divide among older adults functions primarily as a marker of socioeconomic disadvantage rather than an independent driver of stroke risk. Digital inclusion efforts alone are therefore unlikely to reduce stroke incidence without concurrent attention to the underlying social determinants—including educational attainment, income inequality, and equitable access to health care—that simultaneously shape both digital participation and cerebrovascular risk.

At the clinical level, assessing patients’ health-related digital engagement may provide a low-burden indicator of socioeconomic vulnerability during routine evaluation, informing more targeted care planning for older adults at elevated risk. At the health systems level, equitable access requires offering multiple modalities of service delivery—including telephone and in-person options—alongside digital platforms, ensuring that those least likely to engage digitally are not systematically excluded from care.

For public health policy, digital inclusion programs should extend beyond the provision of devices and internet access to encompass health-related digital literacy, age-friendly interface design, and long-term technical support for vulnerable groups. These efforts are most likely to benefit health outcomes when integrated into broader strategies addressing the social determinants of health rather than implemented in isolation.

### Strengths and Limitations

This study has several methodological strengths. First, the primary exposure was operationalized as health-related digital engagement, encompassing online interaction with medical providers, health insurance management, health information seeking, and prescription management, rather than as general internet access. This definition aligns the exposure more closely with mechanisms relevant to health behaviors and chronic disease management. Second, the sequential adjustment strategy across 4 nested models transparently delineated the confounding structure and quantified the contribution of socioeconomic factors to the observed association. Third, the use of a nationally representative longitudinal cohort with up to 9 years of follow-up, survey-weighted discrete-time hazard models fitted to wave-structured data, and incorporation of NHATS sampling weights, primary sampling units, and strata strengthened the analytical rigor and generalizability of the findings.

Several limitations warrant consideration. First, residual confounding by unmeasured factors, including detailed cognitive function, quality of social support, health literacy, and regional heterogeneity in health care access, cannot be excluded. Second, the HDEI, several covariates, and stroke history were derived from self-report or proxy report, and stroke ascertainment in NHATS was not adjudicated against medical records or imaging. Although previous validation studies have reported high sensitivity and specificity for self-reported stroke diagnoses [36,37], any remaining nondifferential misclassification of the exposure or outcome would likely bias estimates toward the null. The HDEI also assumes equal weighting of 4 binary items. One item, online prescription ordering or refilling, used a past-month rather than a past-year recall window; this difference may affect detection sensitivity but not the item weight.

Third, death from nonstroke causes is a competing risk over the 9-year follow-up. The primary cause-specific discrete-time hazard model treated deaths before stroke as censoring. A prespecified discrete-time competing-risks analysis using multinomial logistic regression, with death ascertained from NHATS Tracker File round-specific status variables, yielded a model 3 relative risk ratio for stroke of 0.92 (95% CI 0.79‐1.07; *P*=.27), closely matching the primary discrete-time model 3 estimate (Multimedia Appendix 7). Nevertheless, fine-gray subdistribution hazard modeling using continuous-time death dates would be a useful extension if month-level mortality data become available.

Fourth, 2 structural features of the data warrant caution. Household income was missing for approximately 42% (2270/5384) of the analytic sample. This issue was addressed using a missing-indicator approach in the primary models and MICE in sensitivity analyses. However, the missing-indicator approach grouped participants with missing, “don’t know,” or refused income responses into a single heterogeneous category, despite potential differences in economic resources, reporting preferences, health literacy, and health status. This grouping may introduce residual socioeconomic confounding, and the accompanying MICE analysis cannot fully correct for potential missing-not-at-random mechanisms. Although IPTW was used to address covariate imbalance across HDEI groups, postweighting SMDs remained above 0.10 for several variables, including age group, race or ethnicity, educational attainment, household income, ADL disability, social isolation, and cell phone use. This finding indicates incomplete covariate balance and possible violations of positivity. Therefore, the IPTW results should be interpreted as supportive rather than definitive, and residual socioeconomic confounding may remain despite weighting. Finally, HDEI was measured only at Wave 1 and treated as time fixed. Given the rapid evolution of digital technology between 2011 and 2020, exposure drift may have attenuated the observed associations, and future studies should consider time-varying measures of digital engagement.

### Conclusions

In this nationally representative cohort of older Medicare beneficiaries, greater health-related digital engagement was associated with a lower risk of incident stroke in unadjusted and demographically adjusted models. However, this association was substantially attenuated and was no longer statistically significant after adjustment for socioeconomic factors. These findings are consistent with socioeconomic confounding and suggest that health-related digital engagement may serve, at least in part, as a marker of broader socioeconomic advantage. Future studies should further evaluate whether digital engagement has an independent causal role in stroke prevention and more explicitly address the structural determinants of both digital access and cerebrovascular risk.

## Supplementary material

10.2196/93631 Multimedia Appendix 1 Directed acyclic graph illustrating the hypothesized causal structure between health-related digital engagement and incident stroke.

10.2196/93631 Multimedia Appendix 2 Attrition analysis: baseline comparison of retained and excluded participants.

10.2196/93631 Multimedia Appendix 3 Variance inflation factor diagnostics.

10.2196/93631 Multimedia Appendix 4 Sensitivity analyses for the association between the Health-Related Digital Engagement Index and incident stroke.

10.2196/93631 Multimedia Appendix 5 Multiple imputation sensitivity analysis for missing household income data.

10.2196/93631 Multimedia Appendix 6 Inverse probability of treatment weighting sensitivity analysis using survey-weighted marginal structural discrete-time hazard models.

10.2196/93631 Multimedia Appendix 7 Discrete-time competing-risks sensitivity analysis (death as competing event).

10.2196/93631 Multimedia Appendix 8 Sensitivity analysis: disaggregated chronic-disease covariates (Model 4b).

10.2196/93631 Multimedia Appendix 9 Sensitivity analysis: exclusion of participants with baseline dementia.

## Abbreviations

ADL: activities of daily living
DAG: directed acyclic graph
HDEI: Health-Related Digital Engagement Index
HR: hazard ratio
IPTW: inverse probability of treatment weighting
IRB: Institutional Review Board
MICE: multiple imputation by chained equations
NHATS: National Health and Aging Trends Study
SMD: standardized mean difference
SP: Sample Person
VIF: variance inflation factor
