# Supplementary material for: Health-Related Digital Engagement and Incident Stroke Among Older Adults: Prospective Cohort Study
Source: J Med Internet Res. 2026 Jul 6;28:e93631. doi: 10.2196/93631 (PMC13336533; doi:10.2196/93631)

**Figure S1.** Directed acyclic graph illustrating the hypothesized causal structure between health-related digital engagement and incident stroke.

HDEI indicates the Health-Related Digital Engagement Index. The blue node represents the exposure, and the red node represents the outcome. Demographic covariates, including age and sex, and socioeconomic covariates, including race or ethnicity, education, and household income, were conceptualized as measured confounders and considered as the primary adjustment set. Baseline health and social-context factors, including chronic disease, ADL disability, and social isolation, were additionally considered in an extended model to evaluate the robustness of the association. Solid arrows indicate the hypothesized causal path of substantive interest, dashed arrows indicate back-door confounding paths, and dotted arrows indicate additional baseline health or social-context pathways. Potential effect modification by age, sex, race or ethnicity, and education was evaluated separately in prespecified subgroup and interaction analyses. No collider or post-exposure variable was intentionally adjusted for in the primary model. Arrows among covariates are omitted for clarity; the structure permits arbitrary correlations within covariate blocks.


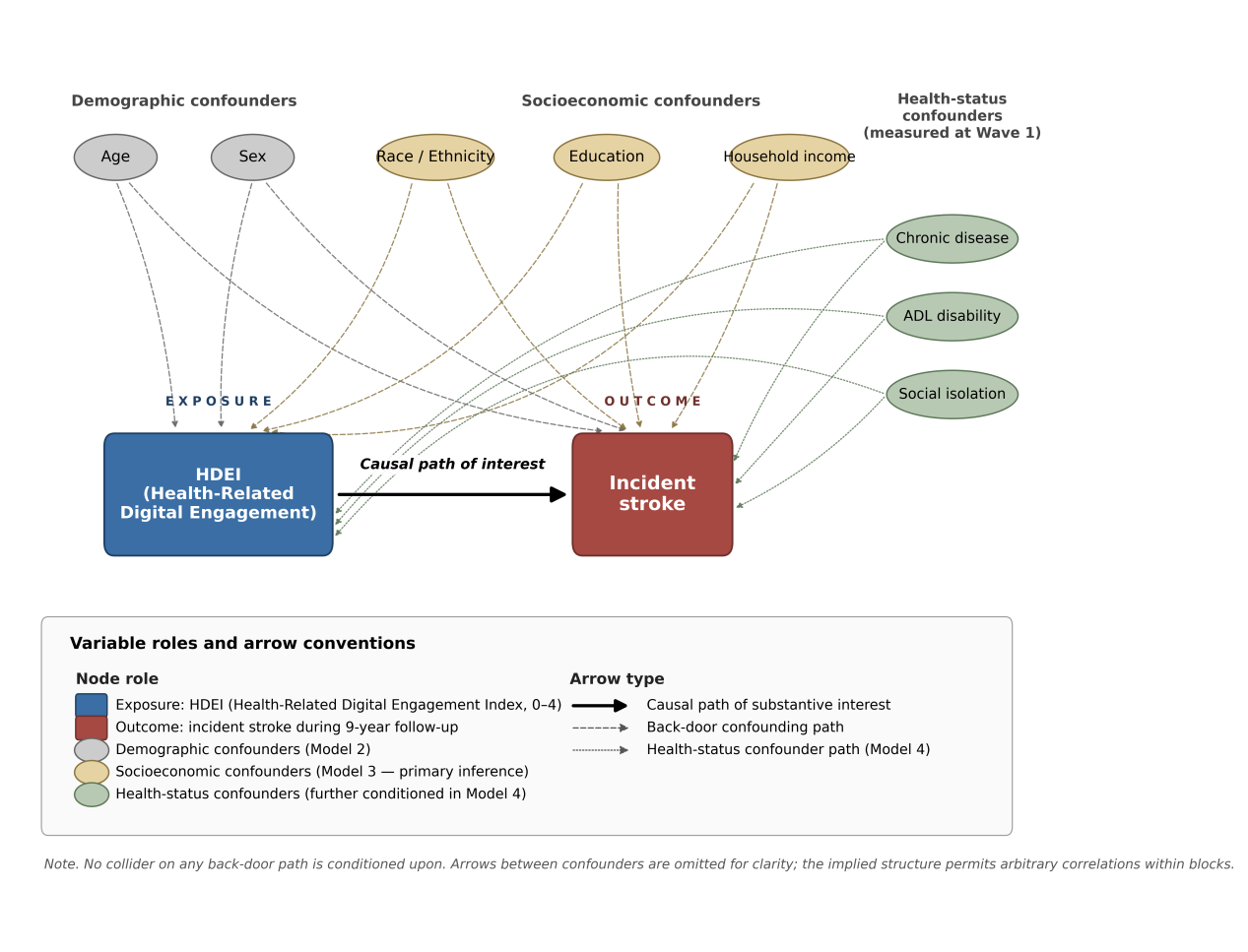

Supplement: Multimedia Appendix 1 [file jmir-v28-e93631-s001.docx]
