# Supplementary material for: Health-Related Digital Engagement and Incident Stroke Among Older Adults: Prospective Cohort Study
Source: J Med Internet Res. 2026 Jul 6;28:e93631. doi: 10.2196/93631 (PMC13336533; doi:10.2196/93631)
Supplement: Multimedia Appendix 2 [file jmir-v28-e93631-s002.docx]

**Table S2.** Baseline characteristics of participants retained in the analytic cohort and those excluded due to missing follow-up data, NHATS Wave 1 (2011).

| **Characteristic** | **Retained (n = 5,384)** | **Excluded (n = 1,799)** | **SMD** | **P value** |
| --- | --- | --- | --- | --- |
| ***Age group, n (%)*** | | | | |
| 65-69 years | 1,071 (19.9) | 241 (13.4) | 0.343 | <.001 |
| 70-74 years | 1,155 (21.5) | 307 (17.1) |  |  |
| 75-79 years | 1,097 (20.4) | 316 (17.6) |  |  |
| 80-84 years | 1,028 (19.1) | 338 (18.8) |  |  |
| 85-89 years | 624 (11.6) | 287 (16.0) |  |  |
| >=90 years | 409 (7.6) | 310 (17.2) |  |  |
| ***Sex, n (%)*** | | | | |
| Female | 3,146 (58.4) | 1,127 (62.6) | 0.086 | .002 |
| ***Race or ethnicity, n (%)*** | | | | |
| White, non-Hispanic | 3,738 (69.4) | 1,178 (65.5) | 0.128 | <.001 |
| Black, non-Hispanic | 1,144 (21.2) | 419 (23.3) |  |  |
| Other, non-Hispanic | 143 (2.7) | 59 (3.3) |  |  |
| Hispanic | 312 (5.8) | 111 (6.2) |  |  |
| ***Education, n (%)*** | | | | |
| ≤ High school | 2,779 (51.6) | 785 (43.6) | 0.329 | <.001 |
| Some college | 1,059 (19.7) | 261 (14.5) |  |  |
| Bachelor's or higher | 1,498 (27.8) | 254 (14.1) |  |  |
| Missing | 48 (0.9) | 499 (27.7) |  |  |
| ***Household income, n (%)*** | | | | |
| Missing/DK/RF | 2,270 (42.2) | 1,158 (64.4) | 0.457 | <.001 |
| <$25,000 | 1,374 (25.5) | 337 (18.7) |  |  |
| $25,000-$49,999 | 808 (15.0) | 165 (9.2) |  |  |
| $50,000-$74,999 | 421 (7.8) | 82 (4.6) |  |  |
| >=$75,000 | 511 (9.5) | 57 (3.2) |  |  |
| ***HDEI score*** | | | | |
| Mean (SD) | 0.31 (0.76) | 0.14 (0.52) | 0.264 | <.001 |
| Score = 0, n (%) | 4,395 (81.6) | 1,649 (91.7) |  |  |
| Score = 1, n (%) | 566 (10.5) | 104 (5.8) |  |  |
| Score >= 2, n (%) | 423 (7.9) | 46 (2.6) |  |  |
| ***Health status indices, mean (SD)*** | | | | |
| Chronic disease burden | 2.37 (1.46) | 1.83 (1.70) | 0.343 | <.001 |
| ADL disability index | 0.83 (1.48) | 0.90 (1.69) | 0.045 | .086 |
| Social isolation index | 2.65 (1.21) | 2.21 (1.65) | 0.305 | <.001 |
| ***Technology use, n (%)*** | | | | |
| Cellphone use | 3,959 (73.5) | 883 (49.1) | 0.519 | <.001 |

**Notes.** Participants with missing HDEI data (n = 170) and those with baseline stroke (n = 892) were excluded prior to this comparison. The exclusion of n = 1,799 refers specifically to participants without any follow-up interview data from Wave 2 through Wave 10. Categorical variables are reported as n (%); continuous variables as mean (SD). Standardized mean differences (SMD) > 0.10 indicate meaningful imbalance. SMD and P values for categorical variables are reported for the overall distribution; P values for continuous variables were derived from independent-samples t-tests. P values for categorical variables were derived from chi-squared tests. Blank cells indicate the SMD and P value are shared with the first row of the category.
