# Supplementary material for: Health-Related Digital Engagement and Incident Stroke Among Older Adults: Prospective Cohort Study
Source: J Med Internet Res. 2026 Jul 6;28:e93631. doi: 10.2196/93631 (PMC13336533; doi:10.2196/93631)
Supplement: Multimedia Appendix 3 [file jmir-v28-e93631-s003.docx]

**Table S5.** Variance Inflation Factors for Models 2, 3, and 4

| **Covariate** | **Model 2 (demographic)** | **Model 3 (+ SES)** | **Model 4 (fully adjusted)** |
| --- | --- | --- | --- |
| HDEI (continuous, 0–4) | 1.05 | 1.19 | 1.20 |
| Age 70–74 (ref: 65–69) | 1.65 | 1.66 | 1.66 |
| Age 75–79 | 1.64 | 1.66 | 1.68 |
| Age 80–84 | 1.63 | 1.65 | 1.67 |
| Age 85–89 | 1.43 | 1.46 | 1.48 |
| Age ≥90 | 1.32 | 1.35 | 1.39 |
| Female | 1.02 | 1.07 | 1.11 |
| Black, non-Hispanic | — | 1.09 | 1.10 |
| Other, non-Hispanic | — | 1.03 | 1.03 |
| Hispanic | — | 1.06 | 1.07 |
| Some college | — | 1.19 | 1.20 |
| Bachelor's degree or above | — | 1.41 | 1.44 |
| Income missing/DK/RF | — | 1.61 | 1.63 |
| Income $25,000–$49,999 | — | 1.44 | 1.45 |
| Income $50,000–$74,999 | — | 1.32 | 1.33 |
| Income ≥$75,000 | — | 1.56 | 1.57 |
| ADL disability index | — | — | 1.19 |
| Chronic disease burden | — | — | 1.12 |
| Social isolation index | — | — | 1.09 |
| **Maximum VIF** | **1.65** | **1.66** | **1.68** |

VIF = variance inflation factor. All VIFs < 2.0 indicate acceptable multicollinearity. Dashes (—) indicate the variable was not included in that model. Bold would indicate VIF > 5 (none present).
