# Supplementary material for: Health-Related Digital Engagement and Incident Stroke Among Older Adults: Prospective Cohort Study
Source: J Med Internet Res. 2026 Jul 6;28:e93631. doi: 10.2196/93631 (PMC13336533; doi:10.2196/93631)
Supplement: Multimedia Appendix 4 [file jmir-v28-e93631-s004.docx]

**Table S1.** Sensitivity Analyses for the Association Between HDEI and Incident Stroke

| **Analysis** | **Exposure** | **Model** | **No.** | **Events** | **HR^a^** | **95% CI** | **P value** |
| --- | --- | --- | --- | --- | --- | --- | --- |
| Excluding wave 2 events | HDEI (continuous) | Model 2 | 4,292 | 377 | 0.82 | 0.70–0.95 | 0.010 |
|  |  | Model 4 | 4,250 | 374 | 0.91 | 0.78–1.06 | 0.237 |
| Alternative exposure | Cellphone use (binary) | Model 2 | 5,384 | 472 | 0.77 | 0.62–0.95 | 0.014 |
|  |  | Model 4 | 5,325 | 465 | 0.90 | 0.73–1.12 | 0.345 |

**Abbreviations:** HR, hazard ratio; CI, confidence interval; HDEI, health-related digital engagement index; ADL, activities of daily living.

^a^ HR represents the effect per 1‑unit increase in HDEI (continuous, range 0–4) or the effect of cellphone use (yes vs no).

Two sensitivity analyses were performed to test the robustness of findings. First, events occurring during wave 2 were excluded to minimize reverse causation bias (i.e., participants who experienced a stroke shortly after baseline assessment may have had preclinical disease influencing their digital engagement). Second, cellphone use (binary) was substituted as an alternative technology exposure to assess whether the observed associations were specific to health‑related digital engagement or reflected a nonspecific technology‑use effect. Model 2 was adjusted for age and sex. Model 4 was additionally adjusted for race or ethnicity, education, household income, chronic disease burden, ADL disability, and social isolation. All models included wave indicator variables. Bold values indicate statistical significance at the .05 level.
