# Supplementary material for: Health-Related Digital Engagement and Incident Stroke Among Older Adults: Prospective Cohort Study
Source: J Med Internet Res. 2026 Jul 6;28:e93631. doi: 10.2196/93631 (PMC13336533; doi:10.2196/93631)
Supplement: Multimedia Appendix 5 [file jmir-v28-e93631-s005.docx]

**Table S3.** Survey-weighted MICE-based sensitivity analysis using discrete-time hazard models

| **Model** | **Exposure contrast** | **Primary survey-weighted HR (95% CI)** | **Complete-case survey-weighted HR (95% CI)** | **MICE-imputed pooled survey-weighted HR (95% CI)** | **P value** |
| --- | --- | --- | --- | --- | --- |
| Model 3 | Per 1-point increase | 0.92 (0.79-1.06) | 0.90 (0.75-1.07) | 0.93 (0.80-1.07) | .302 |
| Model 3 | HDEI score 1 vs 0 | 0.82 (0.58-1.15) | 0.84 (0.55-1.30) | 0.86 (0.61-1.20) | .377 |
| Model 3 | HDEI score ≥2 vs 0 | 0.93 (0.63-1.37) | 0.90 (0.56-1.45) | 0.95 (0.65-1.40) | .806 |
| Model 4 | Per 1-point increase | 0.91 (0.79-1.05) | 0.90 (0.75-1.07) | 0.92 (0.80-1.06) | .272 |
| Model 4 | HDEI score 1 vs 0 | 0.83 (0.59-1.16) | 0.85 (0.55-1.31) | 0.87 (0.62-1.21) | .400 |
| Model 4 | HDEI score ≥2 vs 0 | 0.93 (0.63-1.36) | 0.89 (0.55-1.43) | 0.94 (0.64-1.39) | .766 |

**Note.** Hazard ratios were estimated using survey-weighted discrete-time hazard models with a complementary log-log link fitted to person-period data. Survey design was specified using NHATS sampling weights, primary sampling units, and strata in each completed dataset. Missing baseline covariates were imputed using multiple imputation by chained equations. Each completed dataset was transformed into the same person-period structure used in the primary analysis. Estimates were pooled on the log-hazard-ratio scale using Rubin’s rules and exponentiated to obtain pooled HRs and 95% CIs. The complete-case column is restricted to 3105 participants with observed household income and covariates; the MICE column uses the full analytic cohort of 5384 participants. Household income, education, and race/ethnicity were missing in 42.2%, 0.9%, and 0.9% of participants, respectively. CI, confidence interval; HDEI, Health-Related Digital Engagement Index; HR, hazard ratio; MICE, multiple imputation by chained equations; NHATS, National Health and Aging Trends Study.
