# Supplementary material for: Health-Related Digital Engagement and Incident Stroke Among Older Adults: Prospective Cohort Study
Source: J Med Internet Res. 2026 Jul 6;28:e93631. doi: 10.2196/93631 (PMC13336533; doi:10.2196/93631)
Supplement: Multimedia Appendix 6 [file jmir-v28-e93631-s006.docx]

**Table S4A.** IPTW outcome model results

| **Model** | **Exposure contrast** | **HR (95% CI)** | **P value** |
| --- | --- | --- | --- |
| IPTW marginal structural discrete-time hazard model | Per 1-point increase in HDEI | 0.84 (0.71-1.00) | .048 |
| IPTW marginal structural discrete-time hazard model | HDEI score 1 vs 0 | 0.82 (0.54-1.24) | .330 |
| IPTW marginal structural discrete-time hazard model | HDEI score 2 or higher vs 0 | 0.68 (0.42-1.09) | .108 |
| Doubly robust IPTW model | Per 1-point increase in HDEI | 0.86 (0.73-1.03) | .096 |

**Note.** Survey-weighted marginal structural discrete-time hazard models with stabilized IPTW from multinomial HDEI propensity scores (99th-percentile trimming), combined with NHATS Wave 1 weights, strata, and PSUs. The doubly robust model also adjusted for baseline covariates.

**Table S4B.** Covariate balance before and after IPTW

| **Covariate** | **Pre-IPTW maximum \|SMD\|** | **Post-IPTW maximum \|SMD\|** | **Balanced after IPTW** |
| --- | --- | --- | --- |
| Age group | 0.390 | 0.243 | No |
| Sex | 0.301 | 0.066 | Yes |
| Race/ethnicity | 0.401 | 0.211 | No |
| Educational attainment | 0.815 | 0.175 | No |
| Household income | 0.626 | 0.124 | No |
| Chronic disease burden | 0.086 | 0.051 | Yes |
| ADL disability | 0.319 | 0.185 | No |
| Social isolation | 0.388 | 0.126 | No |
| Cellphone use | 0.699 | 0.627 | No |

**Note.** Covariate balance was assessed using the maximum absolute standardized mean difference (SMD) across pairwise HDEI group contrasts. Values <0.10 indicate acceptable balance. Differences between Table 1 and Table S4B reflect different SMD calculation methods (descriptive pairwise vs IPTW-based balance diagnostics) rather than inconsistencies in the underlying data.
