# Supplementary material for: Health-Related Digital Engagement and Incident Stroke Among Older Adults: Prospective Cohort Study
Source: J Med Internet Res. 2026 Jul 6;28:e93631. doi: 10.2196/93631 (PMC13336533; doi:10.2196/93631)
Supplement: Multimedia Appendix 7 [file jmir-v28-e93631-s007.docx]

**Table S6.** Primary discrete-time hazard model versus discrete-time competing-risks analysis with death as a competing event

| **Model** | **Cause-specific discrete-time HR for stroke (95% CI), P** | **Competing-risks stroke RRR (95% CI), P** |
| --- | --- | --- |
| Model 1 | 0.76 (0.66–0.88), <.001 | 0.78 (0.68–0.90), .001 |
| Model 2 | 0.84 (0.72–0.96), .012 | 0.84 (0.72–0.97), .015 |
| Model 3 (primary) | 0.92 (0.79–1.06), .228 | 0.92 (0.79–1.07), .27 |

Estimates are per 1-point increase in HDEI (continuous, range 0–4). The competing event is death ascertained from NHATS Tracker File round-specific status variables (r2status–r10status). Non-death loss to follow-up is treated as non-informative censoring at the last completed wave. CI, confidence interval; HR, hazard ratio; RRR, relative risk ratio.
