# Supplementary material for: Health-Related Digital Engagement and Incident Stroke Among Older Adults: Prospective Cohort Study
Source: J Med Internet Res. 2026 Jul 6;28:e93631. doi: 10.2196/93631 (PMC13336533; doi:10.2196/93631)
Supplement: Multimedia Appendix 8 [file jmir-v28-e93631-s008.docx]

**Table S7.** HDEI hazard ratio per 1-point increase across alternative chronic-disease parameterisations.

| **Model** | **HDEI HR per 1-point (95% CI), P** | **n** |
| --- | --- | --- |
| Model 3 (primary inferential framework: demographic + socioeconomic adjustment) | 0.92 (0.79–1.06), .228 | 5,325 |
| Model 4 (Model 3 + chronic-disease summary index 0–9 + ADL + social isolation; published version) | 0.91 (0.79–1.05), .211 | 5,325 |
| Model 4b (Model 3 + 9 individual chronic-disease indicators + ADL + social isolation; this Appendix) | 0.91 (0.79–1.05), .180 | 5,325 |
