# Supplementary material for: Health-Related Digital Engagement and Incident Stroke Among Older Adults: Prospective Cohort Study
Source: J Med Internet Res. 2026 Jul 6;28:e93631. doi: 10.2196/93631 (PMC13336533; doi:10.2196/93631)
Supplement: Multimedia Appendix 9 [file jmir-v28-e93631-s009.docx]

**Table S8.** HDEI hazard ratio per 1-point increase: full analytic sample versus dementia-free sub-sample

| **Model** | Full sample estimate | Dementia-free estimate |
| --- | --- | --- |
| Model 1 (unadjusted) | 0.76 (0.66–0.88), .0006 n=5,384; 472 events | 0.77 (0.66–0.89), .001 n=5,169; 442 events |
| Model 2 (+ age, sex) | 0.84 (0.72–0.96), .0121 n=5,384; 472 events | 0.83 (0.72–0.96), .013 n=5,169; 442 events |
| Model 3 (+ race or ethnicity, education, income; primary) | 0.92 (0.79–1.06), .2278 n=5,325; 465 events | 0.91 (0.78–1.06), .200 n=5,118; 436 events |

Hazard ratios are per 1-point increase in HDEI (continuous, range 0–4). Model 1: unadjusted with wave indicators only. Model 2: + age (six categories) and sex. Model 3 (primary inferential framework): + race or ethnicity, educational attainment, and household income (missing income retained as a separate category). Baseline dementia is operationalised as self-reported physician-diagnosed dementia at the Wave 1 interview.
